# In female rats, ethylene glycol treatment elevates protein expression of hepatic and renal oxalate transporter sat-1 (Slc26a1) without inducing hyperoxaluria

**DOI:** 10.3325/cmj.2015.56.447

**Published:** 2015-10

**Authors:** Davorka Breljak, Hrvoje Brzica, Ivana Vrhovac, Vedran Micek, Dean Karaica, Marija Ljubojević, Ankica Sekovanić, Jasna Jurasović, Dubravka Rašić, Maja Peraica, Mila Lovrić, Nina Schnedler, Maja Henjakovic, Waja Wegner, Gerhard Burckhardt, Birgitta C. Burckhardt, Ivan Sabolić

**Affiliations:** 1 Molecular Toxicology, Institute for Medical Research and Occupational Health, Zagreb, Croatia; 2 Analytical Toxicology & Mineral Metabolism, Institute for Medical Research and Occupational Health, Zagreb, Croatia; 3 Toxicology Units, Institute for Medical Research and Occupational Health, Zagreb, Croatia; 4 Clinical Institute of Laboratory Diagnosis, University Hospital Center, Zagreb, Croatia; 5 Systemic Physiology and Pathophysiology, University Medical Center Göttingen, Göttingen, Germany

## Abstract

**Aim:**

To investigate whether the sex-dependent expression of hepatic and renal oxalate transporter sat-1 (Slc26a1) changes in a rat model of ethylene glycol (EG)-induced hyperoxaluria.

**Methods:**

Rats were given tap water (12 males and 12 females; controls) or EG (12 males and 12 females; 0.75% v/v in tap water) for one month. Oxaluric state was confirmed by biochemical parameters in blood plasma, urine, and tissues. Expression of sat-1 and rate-limiting enzymes of oxalate synthesis, alcohol dehydrogenase 1 (Adh1) and hydroxy-acid oxidase 1 (Hao1), was determined by immunocytochemistry (protein) and/or real time reverse transcription polymerase chain reaction (mRNA).

**Results:**

EG-treated males had significantly higher (in μmol/L; mean ± standard deviation) plasma (59.7 ± 27.2 vs 12.9 ± 4.1, *P* < 0.001) and urine (3716 ± 1726 vs 241 ± 204, *P* < 0.001) oxalate levels, and more abundant oxalate crystaluria than controls, while the liver and kidney sat-1 protein and mRNA expression did not differ significantly between these groups. EG-treated females, in comparison with controls had significantly higher (in μmol/L) serum oxalate levels (18.8 ± 2.9 vs 11.6 ± 4.9, *P* < 0.001), unchanged urine oxalate levels, low oxalate crystaluria, and significantly higher expression (in relative fluorescence units) of the liver (1.59 ± 0.61 vs 0.56 ± 0.39, *P* = 0.006) and kidney (1.77 ± 0.42 vs 0.69 ± 0.27, *P* < 0.001) sat-1 protein, but not mRNA. The mRNA expression of *Adh1* was female-dominant and that of *Hao1* male-dominant, but both were unaffected by EG treatment.

**Conclusions:**

An increased expression of hepatic and renal oxalate transporting protein sat-1 in EG-treated female rats could protect from hyperoxaluria and oxalate urolithiasis.

Hyperoxaluria is one of the main causes of stone formation in the urinary tract, and results in urine oversaturation with calcium oxalate. It can be induced by an excess of dietary oxalate intake or develop secondary to wide range of disorders (1-3). Urinary oxalate excretion and incidence of urolithiasis in humans and rodents are sex-related, occurring 2-3-times more often in males, but the reasons for this are not quite clear (4-12).

Exogenous oxalate is absorbed from food by paracellular diffusion and transcellularly by secondary-active transporters of the SLC26 family (13,14). Endogenous oxalate is mainly produced in hepatocytes (15-17) and extruded across the sinusoidal membrane in exchange for sulfate by the sulfate anion transporter sat-1 (Slc26a1) (18,19). It is eliminated from the body partially by secretion into the intestine and largely by excretion in the kidneys (3,20). In renal proximal tubules, oxalate is freely filtered, partially reabsorbed, and additionally secreted into the tubule fluid, resulting in a fractional excretion of 120%-130% (21). Renal secretion of oxalate includes sat-1-mediated uptake across the basolateral membrane (BLM) and release across the brush-border membrane (BBM) into the tubule fluid by the chloride-formate exchanger CFEX (Slc26a6) and/or the diastrophic dysplasia sulfate transporter DTDST (Slc26a2) (3,18,19,22). Mice lacking sat-1 or CFEX develop hyperoxalemia, hyperoxaluria, calcium-oxalate nephrocalcinosis, and tubular damage (9,23,24).

The role of sat-1 in development of sex-related urolithiasis has not been well investigated in in vivo animal models. In rats, hepatic and renal sat-1 expression was higher in males than in females (19), which may contribute to a higher prevalence of urolithiasis in males. Also, in male rats, increased hepatic and renal sat-1 expression were not associated with hyperoxaluria (25), while in female rats this issue has not been investigated.

Here we used the existing rat model of experimental hyperoxaluria, in which female and male animals are treated with an oxalate precursor, ethylene glycol (EG) (26-28) in order to test if this pathophysiological condition changes protein expression of hepatic and renal oxalate transporter sat-1 (Slc26a1). In these organs of both sexes, we analyzed the mRNA and protein expression of sat-1, and mRNA expression of the two rate-limiting enzymes of EG metabolism, alcohol dehydrogenase 1 (*Adh1*; EC 1.1.1.1) and hydroxyacid oxidase 1 (*Hao1*; EC 1.1.3.15).

## Material and methods

### Animals and treatment

We used 24 male and 24 female three**-**month old Wistar rats from the breeding colony at the Institute for Medical Research and Occupational Health in Zagreb. The experiments were conducted during 2012 and 2013. Animal care conformed to the institutional regulations, which comply with international guidelines (29). Experiments were approved by the Institutional Ethics Committee. Animals were provided with standard pelleted food (Mucedola, Settimo Milanese, Italy) and either tap water (controls; 12 female and 12 male animals) or EG in tap water (0.75%, vol/vol; changed daily; EG-treated 12 female and 12 male animals) for one month.

### Sampling of urine, blood, and organs

Two days before sacrifice, rats were placed in individual metabolic cages with free access to water (control animals) or EG solution (EG-treated animals), but without food. Urine was collected for 24 hours. Body mass and the volume of each animal’s urine was measured. Urine was centrifuged at 2500 g for 15 min. In the supernatant, biochemical parameters were determined. The pellet was resuspended in 1 mL of the supernatant for testing the presence of oxalate crystals by light microscopy under phase contrast.

On the 31st day after the start of EG-treatment, rats were anaesthetized (Narketan, 80 mg/kg b.m and Xylapan, 12 mg/kg b.m., i.p.), 4-5 mL blood was collected from the heart by percutaneous puncture using heparinized vacutainer, and centrifuged (2500 g for 10 min) to obtain plasma. The animals were bled out, and the liver and kidneys were sampled. These procedures were performed between 8 and 12 am

### Determination of biochemical parameters

Plasma urea levels and urine creatinine and calcium levels were measured in native samples with the automated analyzer Cobas c501 (Roche, Hellerup, Germany). Aliquots of blood plasma were filtered through Ultra-free-MC Millipore filters (10 kDa cut-off) by centrifugation at 2000 g for 30 min to remove proteins. Ultrafiltrate and aliquots of urine were acidified by 1 N HCl, and oxalate and citrate levels were measured by ion chromatography with a conductivity detector (Dionex, Sunyvale, CA, USA) (30,31). The lipid peroxidation product malondialdehyde (MDA) in blood plasma, urine, and liver and kidney tissue homogenates was measured by high pressure liquid chromatography (HPLC) (32-34), whereas reduced glutathione (GSH) in blood plasma and liver and kidney tissue homogenates was analyzed spectrophotometrically (35). Chemical elements in urine samples (Ca, Mg, P, Na, K, S, I, Sr, Se, Fe, Cu, Zn, Co, B, As, Ba, and Li) were analyzed using the inductively-coupled plasma mass spectrometry (IC-PMS) (36). Urine protein concentration was measured by the Bradford assay (37). MDA and GSH were obtained from 6, and all other biochemical parameters from 10-12 animals from each group.

### Antibodies, chemicals, and other material

Monoclonal antibodies for rat sat-1 (sat-1-Ab) and α-actin (actin-Ab), and polyclonal antibody for cell adhesion molecule 105 (CAM105-Ab) were used as previously described (18,19,22,38). CY3-labeled secondary antibodies (donkey anti-mouse [DAMCY3] and goat anti-rabbit [GARCY3] IgG) were purchased from Jackson ImmunoResearch (West Grove, PA, USA). Narketan and Xylapan were purchased from Chassot (Bern, Switzerland) and EG from Merck (Darmstadt, Germany). Other chemicals were of the highest available purity and purchased from either Sigma-Aldrich (St. Louis, MO, USA) or Fisher Scientific (Pittsburgh, PA, USA).

### Tissue fixation, immunocytochemistry, and determination of fluorescence pixel intensity

Tissue fixation with 4% p-formaldehyde in animals in vivo, cryosectioning, optimal antigen retrieval, and immunostaining protocols for sat-1, actin, and CAM105, and fluorescence microscopy were performed as previously described (19,38,39). Immunostaining was analyzed with Opton III RS fluorescence microscope (Opton Feintechnik, Oberkochen, Germany). The images were taken with digital Spot RT slider camera and software (Diagnostic Instruments, Sterling Heights, MI, USA), and processed in Adobe Photoshop 6.0 (Adobe Systems Inc., San Jose, CA, USA). The software settings were adjusted to the strongest fluorescence intensity in a set of cryosections, and all the images were taken using the same settings.

The intensity of CY3 fluorescence (pixel intensity) was measured in the original images using the Image-J software, version 1.4 (NIH, Bethesda, MD, USA). Our previous studies on sat-1-Ab and other antibodies showed a good correlation between the abundance of protein in isolated membranes, represented by the protein band density in Western blots, and fluorescence pixel intensity in immunocytochemical images (19,40,41). Accordingly, in this study only pixel intensity was determined.

A single tissue cryosection was taken from each liver (6 animals per group) and immunostained for sat-1 or CAM105. Three images of each cryosection were taken using a 40 × objective. In each image, 10-15 randomly chosen staining-positive regions of interest (ROI) (sat-1-positive sinusoidal membrane or CAM105-positive canalicular membrane) were circled, fluorescence pixel intensity was measured, averaged, and corrected for the background pixel intensity of unstained cytoplasm. Also from 6 animals per group, one kidney tissue cryosection was taken and immunostained with sat-1-Ab or CAM105-Ab. Three images of each cryosection in the cortex and outer stripe were taken with a 25 × objective. In each image, 5-8 representative ROIs were circled (sat-1-positive BLM in the cortical convoluted tubules or CAM105-positive BBM in the inner stripe tubules), fluorescence pixel intensity was measured, averaged, and corrected for the background staining intensity measured in the unstained cell cytoplasm. The final data were expressed relative to the strongest fluorescence pixel intensity measured in images from the respective control animal.

### RNA isolation, complementary DNA (cDNA) synthesis, and quantitative real-time reverse transcription polymerase chain reaction (qRT-PCR)

After sacrifice, a slice of liver was promptly immersed into RNAlater solution (Sigma). Kidneys were decapsulated and a ~ 1-mm thick middle slice was immediately submerged into the RNAlater solution (Sigma). Total cellular RNA from corresponding tissues ( ~ 50 mg of each organ) was extracted using Trizol (Invitrogen, Karlsruhe, Germany), according to the manufacturer’s instructions. RNA concentration and its purity were estimated by the spectrophotometric measurement of optical density at 260 and 280 nm (BioSpec Nano, Shimadzu, Kyoto, Japan). The integrity of RNA was verified by agarose gel electrophoresis. Isolated RNA was stored at -70°C until further use.

First strand cDNA synthesis was performed by High-Capacity cDNA Reverse Transcription Kits (Applied Biosystems, Foster City, CA, USA) in accordance with the manufacturer’s instructions. Synthesized cDNA was stored at -20°C until use. qRT-PCR was performed in a 50 µL volume using 50 ng of the first strand cDNA template, 2.5 µL of 20 × TaqMan Gene Expression Assays mix, 25 µL of 2 × TaqMan Universal PCR Master Mix (all from Applied Biosystems), and 17.5 µL of nuclease-free water. Primers and probes were designed by Applied Biosystems and supplied as TaqMan Gene Expression Assays mix containing a 20 × mix of unlabeled PCR forward and reverse primers, as well as TaqMan MGB probe. Assay IDs for the rat genes were Rn01637788_m1 (*sat-1*), Rn00570670_m1 (*Adh1*), Rn01486035_m1 (*Hao1*) and Rn00667869_m1 (*β-actin*). Amplification and detection were performed using the 7500 RT-PCR System (Applied Biosystems). Thermal cycling conditions were 2 min at 50°C and 10 min at 95°C, followed by 40 two-step cycles of denaturation (15 sec at 95°C) and annealing/extension (1 min at 60°C). mRNA quantification was performed using TaqMan Assays from Applied Biosystems (available from *http://www.appliedbiosystems.com*) according to manufacturer’s instructions. Nontemplate control reactions, where cDNA was substituted with nuclease-free water, were included in each run to check for possible contamination. To standardize the input of cDNA amount, an endogenous control, ie, the house-keeping gene, β-actin was quantified and results were normalized to these values. Each reaction was performed in duplicate. Quantification of mRNA expression was accomplished by comparative Ct-method using the Relative Quantification Study Software (Applied Biosystems) (42). qRT-PCR study was performed with cDNA obtained from 5 (liver) and 6 (kidneys) independent RNA preparations from control and EG-treated rats of both sexes.

### Statistical analysis

Data are expressed as mean ± standard deviation (SD). The differences between groups were tested with *t* test using STATISTICA software (StatSoft Version 12, Tulsa, OK, USA). The level of statistical significance was set at *P* < 0.05.

## Results

### Effect of EG treatment: biochemical parameters, oxalate crystaluria, and tissue morphology in control and EG-treated female and male animals

Control males had significantly higher body mass, urine volume, urinary protein excretion rate, and urine oxalate relative to creatinine, and significantly lower urine calcium, citrate, magnesium, and phosphate than control females (Table 1). There were no differences between sexes in plasma and urinary creatinine and plasma urea. In comparison with control females, EG-treated females had ~ 60% higher (*P* < 0.001) plasma oxalate; there were no differences in any other plasma parameter. Notably, EG treatment did not increase urinary oxalate excretion. EG-treated males had significantly lower plasma citrate (*P* < 0.001), urine calcium concentration (*P* = 0.002), and calcium excretion rate (*P* < 0.001), significantly higher magnesium excretion rate (*P* = 0.018), and higher plasma oxalate ( ~ 4.6-fold; *P* < 0.001), urine oxalate concentration ( ~ 15-fold; *P* < 0.001), and oxalate excretion rate ( ~ 18-fold; *P* < 0.001) than control males. Other elements determined in urine (Na, K, S, I, Sr, Se, Fe, Cu, Zn, Co, B, As, Ba, and Li) were excreted with a significant variability, exhibiting no major differences between experimental groups of animals (data not shown). MDA and GSH levels showed inconsistent results, suggesting no differences among groups in oxidative status (data not shown).

**Table 1:** Body mass, urine excretion, and relevant biochemical parameters in blood plasma and urine of control and ethylene glycol (EG)-treated female and male rats (means ± standard deviation)

	Females		Males	
Parameter	control (n = 10-12)	EG-treated (n = 10-12)	control (n = 12)	EG-treated (n = 12)
Body mass (g)	196 ± 17.3	198 ± 10.4	340 ± 20.8^†^	351 ± 24.2
Parameters in blood plasma				
Citrate (ultrafiltrate) (µmol/L)	153 ± 48.5	143 ± 31.2	136 ± 38.1	77 ± 38.1^‡^
Oxalate (ultrafiltrate) (µmol/L)	11.6 ± 4.9	18.8 ± 2.9^†^	12.9 ± 4.1	59.7 ± 27.2^‡^
Creatinine (mmol/L)	29 ± 14.5	33 ± 14.9	38 ± 6.6	43 ± 9.4
Urea (μmol/L)	7.7 ± 2.94	6.4 ± 1.21	6.1 ± 0.48	6.6 ± 0.94
Parameters in urine				
Volume (mL/24 h)	17.3 ± 9.6	24.0 ± 9.5	31.2 ± 12.9^§^	30.6 ± 7.9
Flow (mL/24 h kg b. m.*)	99 ± 55.4	121 ± 48.5	92 ± 34.6	95 ± 31.2
Protein (mg/24 h kg b.m.)	21 ± 8.6	18 ± 7.5	35 ± 16.1^§^	30 ± 21.2
Calcium				
μmol/L	3.7 ± 1.59	2.5 ± 1.87	1.1 ± 0.97^†^	0.09 ± 0.03^ǁ^
nmol/24 h kg b.m.	283 ± 113.6	258 ± 186.7	79 ± 54.7^†^	9.3 ± 7.59^‡^
Citrate				
mmol/L	5.5 ± 2.67	4.4 ± 3.12	3.6 ± 3.74	2.2 ± 0.97
μmol/24 h kg b.m.	409 ± 197.4	435 ± 325.6	229 ± 100.5^¶^	200 ± 114.3
Creatinine				
mmol/L	5.3 ± 2.91	3.7 ± 1.45	4.4 ± 3.43	3.8 ± 1.32
μmol/24 h kg b.m.	360 ± 69.3	371 ± 58.9	302 ± 83.1	334 ± 90.1
Oxalate				
µmol/L	161 ± 90.1	161 ± 83.1	241 ± 204.4	3716 ± 1826^‡^
µmol/24 h kg b.m.	12.5 ± 6.1	20.8 ± 20.0	17.0 ± 7.1	322 ± 135.1^‡^
mmol/mol creatinine	34 ± 13.2	55 ± 54.0	61 ± 28.1^§^	994 ± 391.4^‡^
Magnesium				
mmol/L	8.4 ± 3.45	7.1 ± 2.21	2.7 ± 2.12^†^	5.0 ± 3.07
mmol/24 h kg b.m.	0.71 ± 0.16	0.78 ± 0.16	0.24 ± 0.15^†^	0.46 ± 0.22**
Phosphate				
mmol/L	26 ± 12.8	22 ± 6.3	14 ± 6.9^¶^	16 ± 7.7
mmol/24 h kg b.m.	2.2 ± 0.41	2.4 ± 0.57	1.1 ± 0.41^†^	1.4 ± 0.38

*Body mass (b.m.).

†*P* < 0.001 vs control females.

‡*P* < 0.001 vs control males.

§*P* = 0.007 vs control females.

ǁ*P* = 0.002 vs control males.

¶*P* = 0.011-0.016) vs control females.

**P = 0.018 vs control males. Other comparisons were not statistically significant (*P* > 0.05).

**Figure 1:**
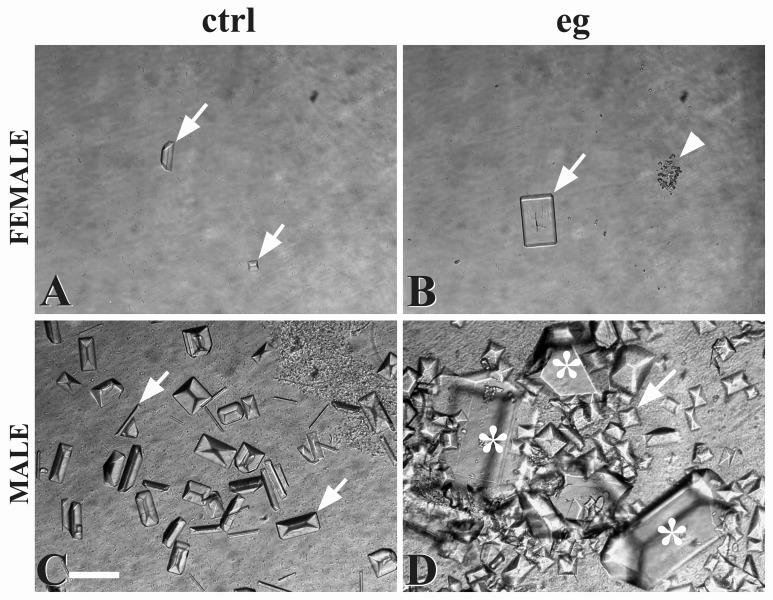
Oxalate crystals in urine sediments of control (ctrl) (**A**) and ethylene glycol (EG)-treated (eg) (**B**) female, and in control (**C**) and EG-treated (**D**) male rats. Prism- and rod-like crystals of various sizes were observed in all samples (arrows), while numerous large crystals were observed in 7 out of 12 EG-treated males (**D**) (asterisks). In 5 EG-treated males, crystaluria was similar to control males. Accumulation of the dust-like crystals was occasionally observed in the sediments from control (not shown) and EG-treated (**B**) animals (arrowheads). Bar, 50 μm.

Control and EG-treated female rats had low abundance of small-sized oxalate crystals in urine sediment (Figures 1A and B). Control males had numerous crystals of heterogeneous size (Figure 1C), whereas 7 out of 12 EG-treated males (60%) had a myriad of crystals, very heterogeneous in size (Figure 1D). Five EG-treated males (40%) had similar number and size of oxalate crystals as controls, in spite of strongly elevated plasma and urine oxalate levels.

**Figure 2:**
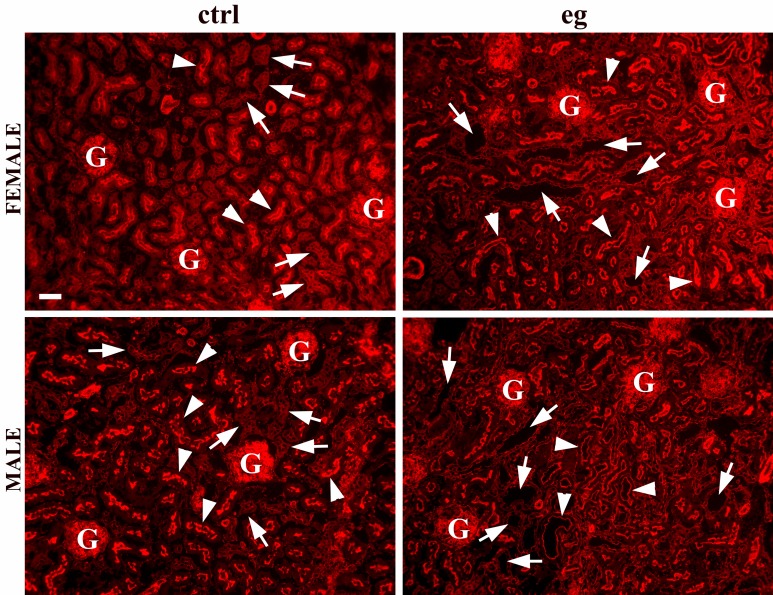
Tissue morphology of the kidney cortex in control (ctrl) and ethylene glycol (EG)-treated (eg) female and male rats in tissue cryosections immunostained with actin-Ab. In the kidneys of control animals of both sexes, we observed regular dense arrangements of undamaged glomeruli (**G**), closed proximal convoluted tubules with thick epithelium and brightly-stained brush-border (arrowheads), and distal tubules and collecting ducts with narrow or closed lumen (arrows). In the kidney cortex of the EG-treated animals of both sexes, distal tubules and collecting ducts had thin epithelium and distended lumen (arrows). In the EG-treated males, many proximal convoluted tubules had wide lumen, thin epithelium, and variable loss of brush border (arrowheads). Images represent findings in 10 animals from each experimental group. Bar, 50 μm.

Tissue morphology in control and EG-treated rats was tested in cryosections immunostained with actin-Ab. The liver tissue of all animals (not shown) and kidney cortex of control females and males (Figure 2) showed regular actin staining and undamaged tissue morphology. In EG-treated females, the overall actin staining was not visibly changed, but many distal tubules and collecting ducts had distended lumina and flattened epithelia. In EG-treated males (Figure 2), most distal tubules and collecting ducts also had distended lumina and flattened epithelium, and some rats had visible loss of actin and damaged proximal tubules.

### Expression of sat-1 protein and mRNA

**Figure 3:**
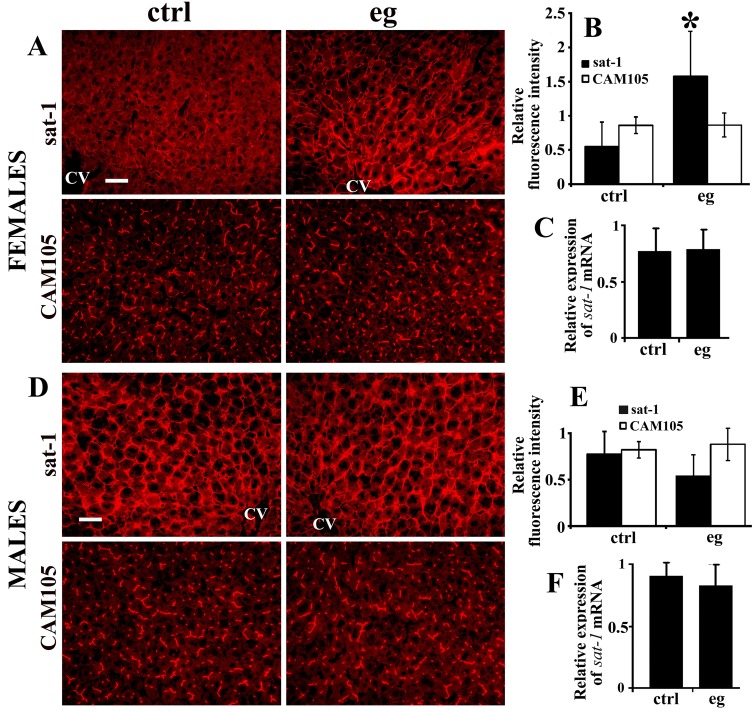
Sulfate anion transporter sat-1 (*Slc26a1*) and cell adhesion molecule 105 (CAM105) protein expression (**A**, **B, D, E**) and *sat-1* mRNA expresion (**C, F**) in the liver of control (ctrl) and EG-treated (eg) rats. (**A-C**) Data in female rats. (**A**) Immunostaining in tissue cryosections. In EG-treated females the intensity of sat-1-related staining of the hepatocyte sinusoidal membrane was stronger, whereas the intensity of CAM105-related staining, localized to both sinusoidal and canalicular membranes, was similar to that in control females. (**B**) Relative fluorescence pixel intensity showed ~ 3-fold increase in the sat-1-related staining intensity in EG-treated females (*vs control, *P* < 0.001; n = 6 in each group) and no significant change for CAM105 (vs control, *P* = 0.828; n = 6 in each group). (**C**) Relative expression of *sat-1* mRNA was similar in control and EG-treated female rats (*P* = 0.939; n = 5 in each group). (**D-F**) Data in male rats. (**D**) Immunostaining in tissue cryosections. In EG-treated males the intensity of sat-1-related staining of the sinusoidal membrane was similar to that in control males, and the same is valid for CAM105. (**E**) Relative fluorescence pixel intensity showed similar staining intensity (*P* > 0.05) in control and EG-treated males for both sat-1 (*P* = 0.496) and CAM105 (*P* = 0.5175) (n = 6 in each group). (**F**) Relative expression of *sat-1* mRNA was similar in control and EG-treated males (*P* = 0.513; n = 5 in each group). CV, central vein. Bars, 20 μm.

Sat-1-Ab-related staining intensity of the hepatocyte sinusoidal membrane in control rats was male-dominant, which is in accordance with our previous findings (19) (Figures 3A and 3D). In control females (Figure 3A), sat-1-Ab weakly stained hepatocytes around the central vein; the staining diminished toward the lobule periphery. In EG-treated females, similar staining pattern was observed, but with ~ 3-fold higher fluorescence intensity (Figures 3A and 3B). The expression of *sat-1* mRNA was similar in the liver tissue of control and EG-treated females (Figure 3C). The pattern of CAM105-Ab-related staining intensity in the canalicular (strong) and sinusoidal (weak) membranes in females was not affected by EG (Figures 3A and 3B). Control and EG-treated males had similar staining intensity of both sat-1 and CAM105 (Figures 3D and 3E), as well as the expression of *sat-1* mRNA (Figure 3F).

**Figure 4:**
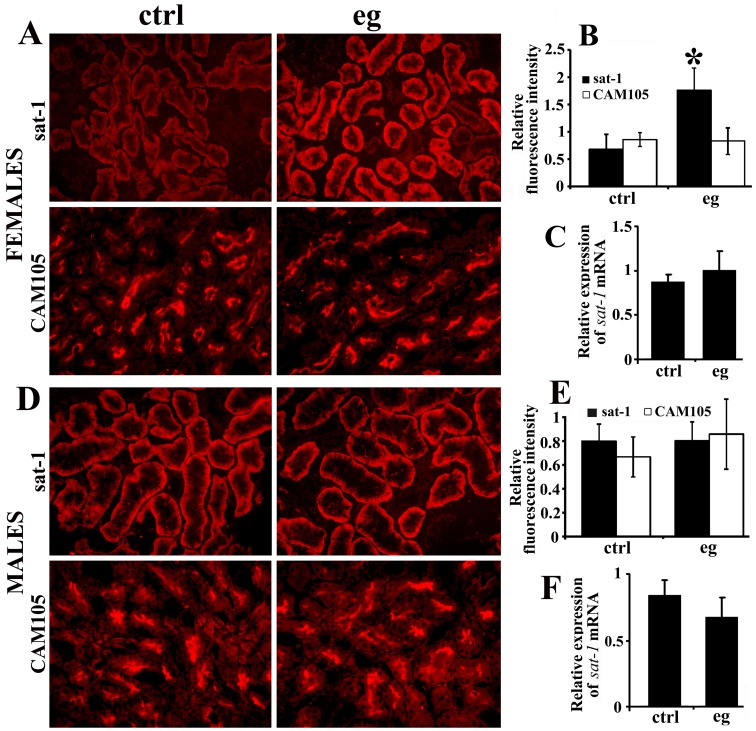
Sulfate anion transporter sat-1 (*Slc26a1*) and cell adhesion molecule 105 (CAM105) protein expression (**A**, **B, D, E**) and *sat-1* mRNA expression (**C, F**) in the kidneys of control (ctrl) and EG-treated (eg) rats. (**A-C**) Data in female rats. (**A**) Immunostaining in tissue cryosections. In EG-treated females the intensity of sat-1-related staining of the basolateral membrane in cortical proximal tubules was stronger than that in controls, whereas the intensity of CAM105-related staining, localized to the proximal tubule brush-border membrane, was similar. (**B**) Relative fluorescence pixel intensity showed ~ 3-fold increase in the sat-1-related staining intensity in EG-treated females (*vs control, *P* < 0.001; n = 6 in each group) and no change for CAM105 (*P* = 0.731; n = 6 in each group). (**C**) Relative expression of *sat-1* mRNA was similar in control and EG-treated female rats (*P* = 0.223; n = 6 in each group). (**D-F**) Data in male rats. (**D**) Immunostaining in tissue cryosections. In EG-treated males the intensity of sat-1-related staining of the basolateral membrane in cortical proximal tubules, and the CAM105-related staining in the proximal tubule brush-border membrane, was similar to that in controls. (**E**) Relative fluorescence pixel intensity showed no differences in the sat-1-related (*P* = 0.927) and CAM105-related (*P* = 0.220) staining intensity between the control and EG-treated males (n = 6 in each group). **(F**) Relative expression of *sat-1* mRNA was similar in control and EG-treated male rats (*P* = 0.125; n = 6 in each group). Bar, 20 μm.

Sat-1-Ab-related staining intensity in the basolateral membrane of cortical proximal tubule cells in control animals was male-dominant, which is in accordance with our previous findings (19) (Figures 4A and 4D). In EG-treated females, the staining intensity of sat-1 increased ~ 3-fold, whereas the CAM105-related staining remained unchanged in both sexes (Figures 4A and 4B, and Figures 4D and 4E, respectively). The expression of sat-1 mRNA in the kidneys was not affected by EG-treatment in either sex (Figures 4C and 4F).

### mRNA expression of the rate-limiting enzymes in EG metabolism

**Figure 5:**
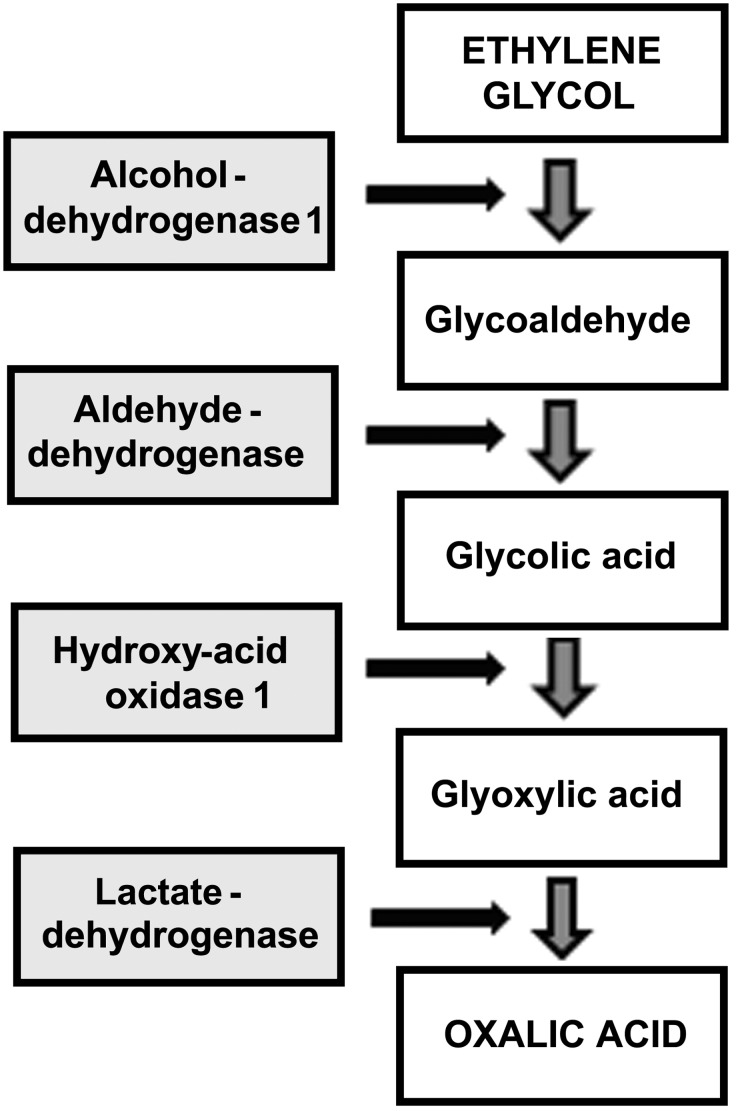
Enzymes that catalyze major steps in metabolic conversion of ethylene glycol (EG) to the end product oxalate in the liver. The first step, conversion of EG to glycoaldehyde, is catalyzed by cytosolic alcohol dehydrogenases 1 (Adh1; EC 1.1.1.1). Glycoaldehyde is metabolized by aldehyde dehydrogenase (EC 1.2.1.3) to glycolate (glycolic acid), which is subsequently metabolized to glyoxylate (glyoxylic acid) by hydroxy-acid oxidase 1 (Hao1; EC 1.1.3.15), and then converted by lactate dehydrogenase (EC 1.1.1.27) to oxalate (oxalic acid). Modified from Williams and Wilson (64) and Hagler and Herman (65).

To estimate if the capacity to produce oxalate in the liver and kidneys was affected by EG-treatment in female and male rats, we determined by qRT-PCR the mRNA expression of two major rate limiting enzymes of EG metabolism, Adh1 and Hao1. Adh1 oxidizes EG to glycoaldehyde, whereas Hao1 catalyzes conversion of glycolic acid to glyoxylic acid (Figure 5).

**Figure 6:**
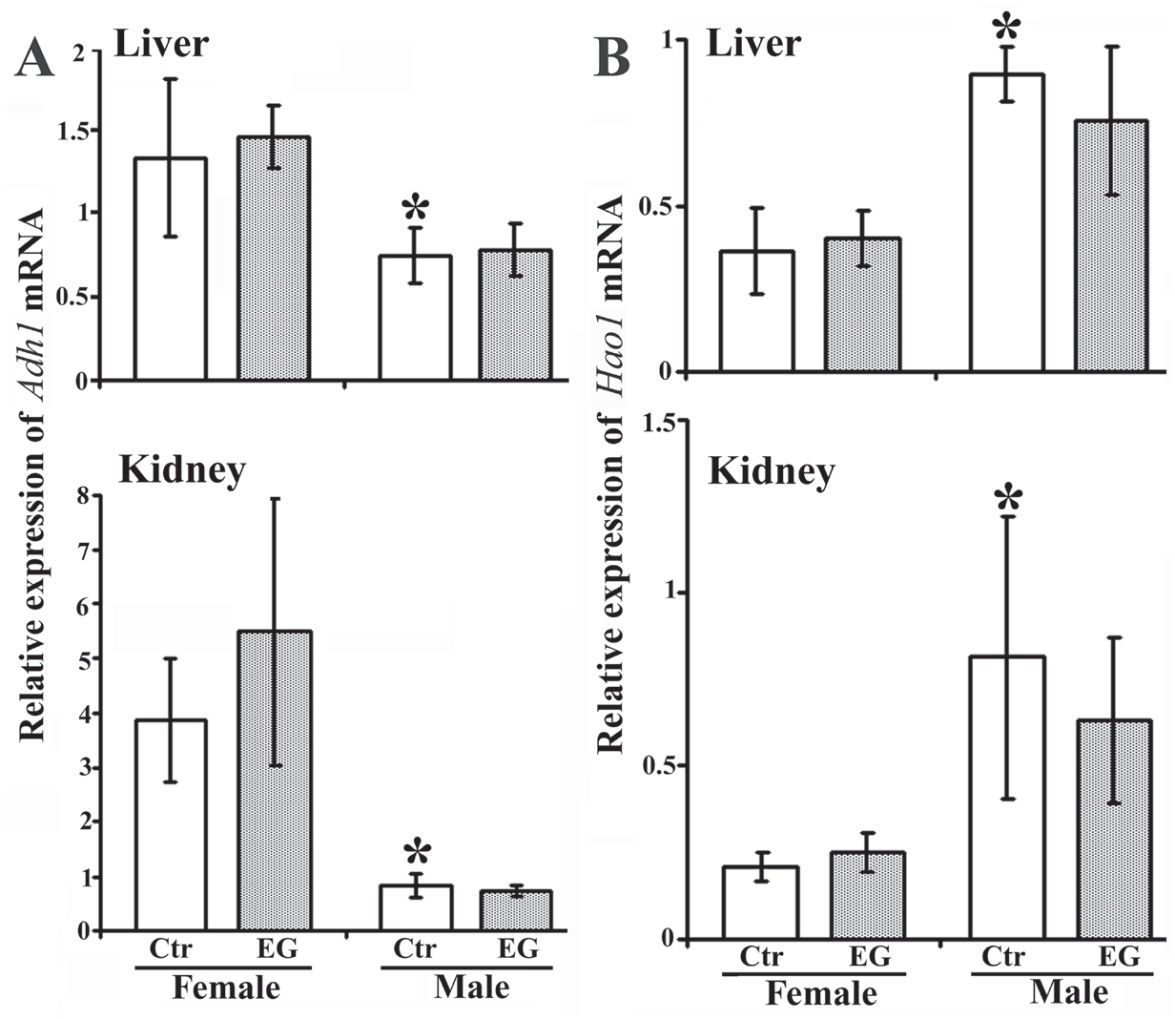
Relative expression of *Adh1* (**A**) and *Hao1* mRNA (**B**) in the liver and kidneys of control (Ctr) and ethylene glycol (EG)-treated (EG) female and male rats. (**A**) In the liver and kidneys of control animals, the relative expression of *Adh1* mRNA in females was respectively ~ 2-fold (*P = 0.028) and ~ 4.3-fold (*P < 0.001) higher than in males, and remained unaffected by EG-treatment in both sexes (vs the respective control data, *P* **≥** 0.127). (**B**) In the liver and kidneys of control animals, the relative expression of *Hao1* mRNA in males was respectively ~ 3-fold (*P < 0.001) and ~ 4-fold (*P < 0.001) higher than in females, and remained unaffected by EG-treatment in both sexes (vs the respective control data, *P***≥**0.167). Bars represent the data from 5 (liver) and 6 (kidney) animals.

In the liver and kidneys of control animals, the relative expression of *Adh1* mRNA in females was respectively ~ 2-fold and ~ 4.5-fold higher than in males, and remained unaffected by EG-treatment in both sexes (Figure 6A). The relative expression of *Hao1* mRNA in the liver and kidneys in control males was respectively ~ 2.5-fold and ~ 4-fold higher than in females and was also unaffected by EG-treatment in both sexes (Figure 6B).

## Discussion

Our study showed hyperoxalemia and hyperoxaluria in EG-treated male rats but surprisingly not in female rats. Non-significant differences in plasma creatinine and urea confirmed an absence of uremia in EG-treated animals. Furthermore, EG-unaffected liver morphology in both sexes indicated that even strong oxalemia, which occurred in EG-treated males, did not change the cell integrity of hepatocytes. However, the kidneys of EG-treated animals exhibited limited morphological damage in several nephron segments and a variable loss of actin. These observations match previous findings (6,12,43-45), which in some studies were attributed to EG-induced oxidative stress (43,46). However, in our study MDA and GSH data indicated no significant oxidative stress in animals.

In control rats, plasma oxalate was similar in both sexes, ~ 12 μmol/L, which corresponds to the values previously published for rodents (7-24 μmol/L) (24,25,27,47,48). In control females, urine oxalate concentration and excretion rate was low, as was the abundance of typical prism- and rod-like oxalate crystals. Oxalate stone formation may have been prevented by higher urine citrate concentration, which complexed calcium ions. In addition, the higher magnesium concentrations may have displaced calcium as binding partner for oxalate (49-53). Control males showed a tendency to have higher urine oxalate concentration and a significantly higher excretion rate than control females. In males, lower urine citrate and magnesium suggest more favorable conditions for crystallization, resulting in an abundance of larger oxalate crystals.

Sex differences in mRNA expression of the rate-limiting enzymes of EG metabolism are in agreement with previous observations in rat liver and kidneys; the female-dominant Adh1 activity and mRNA expression in the liver and kidneys was driven by estrogens (54-57), whereas the male-dominant Hao1 activity and mRNA expression in both organs resulted from androgen stimulation and estrogen inhibition (43,58,59).

EG-treated rats exhibited oxalemia and oxaluria, which is in accordance with previous studies (25,27,28). EG-treated males exhibited low plasma citrate, strong oxalemia ( ~ 4.6-fold above controls), very low urine calcium, hyperoxaluria ( ~ 18-fold above controls), and increased excretion rate of magnesium. The abundance and size of oxalate crystals increased dramatically in most, but not all, EG-treated males although all of them had hyperoxaluria (15-20-fold above controls). Unexpectedly, EG-treated females showed relatively weak oxalemia ( ~ 60% above controls), with other plasma and urine parameters unchanged. They had similar urine oxalate level and the abundance and size of urine oxalate crystals as control females.

It is well-known that testosterone enhances and estrogen inhibits the EG-induced urinary stone formation in rats (6,7), but the pathophysiological processes underlying these differences are unclear. All EG-treated animals drunk the same EG-containing water for one month, but weak oxalemia and absence of oxaluria in females indicates either a much weaker production of oxalate or a much faster elimination of oxalate by some non-renal mechanism, or both.

Regarding oxalate production, we speculate that a) EG should readily cross biological membranes by nonionic diffusion, rendering sex differences in intestinal absorption and availability for metabolism in the liver unlikely; b) females did not ingest less EG-containing water because urine flow in all animal groups was similar, which indicates similar relative uptake and loss of water; c) the relative expression of major EG-metabolizing enzymes, *Adh1* and *Hao1* mRNA in the liver and kidneys was unaffected by EG treatment in both sexes. In accordance with our mRNA findings, Hao1 activity in the liver of male rats was previously found to be unaffected by EG-treatment (58). Future studies need to clarify whether the low expression of Hao1 in female rats was possibly a limiting factor in oxalate production from EG, resulting in the observed modest hyperoxalemia in these animals.

Recent studies in knockout mice revealed that several anion exchangers from the Slc26 family, including sat-1 and CFEX, together controlled oxalate pathways in the intestine (absorption and secretion), liver (production and extrusion), and kidneys (partial reabsorption and secretion). Knockout of sat-1 and CFEX induced oxalemia, hyperoxaluria, and oxalate nephrolithiasis (3,9,20,23,24). These studies concluded that under physiological conditions sat-1 and CFEX contributed to oxalate secretion in the kidneys and intestine, thus protecting from significant oxalemia and oxaluria. In sat-1- and CFEX-deficient mice, the secretory components were diminished, while the absorptive processes in the small intestine prevailed. The enhanced absorption of dietary oxalate causes hyperoxalemia, massive oxalate filtration, hyperoxaluria, and nephrolithiasis (3,20). Thus, these studies identified oxalate transporters from the Slc26 family in the rodent intestine as major contributors to handling of plasma and urine oxalate. However, later studies showed that a) hyperoxaluria in male rats did not alter the renal expression of sat-1 mRNA or protein abundance (25), which is in accordance with our data in EG-treated male rats, and b) sat-1 was not important for oxalate secretion across the mouse duodenum, but its role in oxalate secretion may be confined to more distal parts of the intestine (60).

Our previous study (19) showed mRNA-independent, male-dominant expression of sat-1 protein in the rat liver and kidneys due to testosterone-driven stimulation and estradiol- and progesterone-driven inhibition of its abundance. This study also observed mRNA-independent up-regulation of sat-1 protein expression; while the increase in sat-1-Ab-related staining intensity in both liver and kidneys of EG-treated females corresponded to higher abundance of sat-1 protein, the unchanged expression of mRNA indicated that the observed up-regulation of protein abundance occurred at the posttranscriptional level.

The sat-1 protein via its secretory function may contribute to stronger oxaluria and oxalate crystaluria in male than in female rats. However, in EG-treated males, a high production of oxalate in the liver, and unchanged but high abundance of sat-1 protein in hepatocytes and renal proximal tubules may contribute to hyperoxalemia and hyperoxaluria. It is not known whether sat-1 and other oxalate transporters contribute to oxalate secretion along the intestine of EG-treated males, and their relative expression and activity in control or EG-treated female and male rats has not been determined. Hyperoxalemia and hyperoxaluria in EG-treated males indicate that intestinal secretion of oxalate may be low, possibly due to low male-specific expression of secretory transporters. On the other hand, in the presence of elevated expression of hepatic and renal sat-1 in EG-treated females, a relatively weak oxalemia and absence of hyperoxaluria suggest a possible protective role of the sat-1 protein up-regulation. In hepatocytes, the enhanced abundance of sat-1 protein is possibly caused by a substrate-induced up-regulation in order to eliminate the increased production of oxalate from EG, whereas in the renal proximal tubules, the function of sat-1 protein could be limited by weak oxalemia. Assuming an increased production of oxalate from EG in the liver, weak oxalemia and absence of oxaluria and oxalate crystaluria indicate some compensatory mechanisms that inhibit the crystallization of urine oxalate or stimulate oxalate elimination from the female body. These mechanisms could include changes in concentrations of inhibitors or promotors of calcium-oxalate crystallization in the tubule fluid (52,53). Expression of one of these inhibitors, osteopontin, in rat kidneys was found to be inhibited by testosterone and stimulated by estrogens, and these changes strongly affected oxalate excretion in control and EG-treated female and male rats (61). Additional oxalate secretion could occur in distal intestinal segments, possibly via the EG-induced up-regulated expression of sat-1, CFEX, and other oxalate transporters in the intestinal epithelium. Alternatively, female and male rats could have different abundance of oxalate-degrading bacteria of *Oxalobacter* species, which accelerate oxalate elimination in EG-treated females. These bacteria have been shown to generate a continuous transepithelial gradient for oxalate secretion and ameliorate oxalemia and hyperoxaluria in experimental animals and humans (3,20,62,63). Finally, due to up-regulated expression of the sat-1 in the epithelium, together with abundance of oxalate-degrading bacteria in the intestine, EG-treated female rats could eliminate body oxalate more efficiently, leading to lower oxalemia and absence of significant oxalate crystaluria.

In conclusion, our finding that the expression of hepatic and renal sat-1 protein is stimulated only in EG-treated female rats indicates that a weak hyperoxalemia and absence of hyperoxaluria in these animals may be connected with, and thus may explain, lower incidence of hyperoxaluria and oxalate urolithiasis in female experimental animals and humans. Our findings and theory suffer from technical limitations, and thus should be confirmed by functional studies. Such studies should reveal whether: a) the liver production of oxalate from EG is lower in female than in male rats due to lower expression/activity of Hao1, b) the elimination of oxalate from hepatocytes via elevated sat-1 protein expression is increased in EG-treated females, c) the intestinal excretion of oxalate is higher in EG-treated females, d) the expression of other oxalate-transporting proteins in the liver and kidneys is sex-dependent following EG treatment, and e) the intestinal abundance of oxalate-degrading bacteria is higher in EG-treated females. Our data also indicate that the future studies and possible explanation of pathophysiological conditions that cause the male-dominant hyperoxaluria and oxalate urolithiasis should be shifted from the usual, male-dominant aspects (conditions that generate hyperoxalemia, hyperoxaluria, and oxalate urolithiasis) to the female-dominant aspects (conditions that protect from generation of hyperoxalemia, hyperoxaluria, and oxalate urolithiasis).
